# New horizons: Evolving approaches to uncertainty tolerance scale development

**DOI:** 10.1111/medu.15732

**Published:** 2025-07-01

**Authors:** Michelle D. Lazarus, Melanie Farlie, Md Nazmul Karim

**Affiliations:** ^1^ Centre of Human Anatomy Education (CHAE), Department of Anatomy and Developmental Biology, Biomedicine Discovery Institute, Faculty of Medicine, Nursing, and Health Sciences Monash University Clayton Victoria Australia; ^2^ Monash Centre for Scholarship in Health Education (MCSHE), Faculty of Medicine, Nursing, and Health Sciences Monash University Clayton Victoria Australia; ^3^ Department of Physiotherapy, Faculty of Medicine, Nursing, and Health Sciences Monash University Frankston Victoria Australia; ^4^ School of Public health and preventive Medicine Monash University Melbourne Victoria Australia

## Abstract

Pointing out that learners may feel uneasy in uncertainty yet still respond adaptively, the authors suggest it is time to move on from uncertainty tolerance scales that lack nuance, offering latent trait modeling as an alternative.

The paper titled “Tolerance for uncertainty and medical students' specialty choices: A myth revisited” by Wegwarth and colleagues^1^ provides additional evidence that the existing approaches for uncertainty tolerance scale development and evaluation in health professional learners^2^ and professionals^3^ may not be the optimal approach for measuring this complex construct. In this commentary, we discuss the predominant approaches currently taken in developing and evaluating uncertainty tolerance using scales and suggest ways to evolve these strategies to optimise measurement validity of this construct.

## 1 DEFINING UNCERTAINTY TOLERANCE

Uncertainty tolerance is defined as individuals' perceptions and responses to sources of uncertainty (such as ambiguity, probability or complexity).^4^ An individuals' response to uncertainty is expressed by how they think, feel and act. Uncertainty tolerance construct modelling by Hillen and colleagues (2017)^4^ describes a multi‐component construct comprised of (1) the source of uncertainty, (2) how this source is perceived by an individual and (3) each subsequent response (emotions, thoughts and behaviours).^4^ However, the degree to which these elements contribute to the overall measurement of this construct remains (ironically) uncertain.^2^, ^3^ Further complicating this is the question as to the changeability of one's uncertainty tolerance, which influences measurement approaches. In the mid‐20th Century, uncertainty tolerance was conceptualised as a static personality trait.^4^ Mounting contemporary evidence, however, suggests that uncertainty tolerance is a dynamic and changeable state‐based construct influenced by contextual and personal factors such as prior experiences, geographic location and reflective capacity.^5^

There is evidence that uncertainty tolerance is, at least in part, state‐based in learners. Learners can feel negatively about an experience of uncertainty but can act adaptively (e.g. with uncertainty tolerance) in response through their cognition and behaviour.^6^ For example, a medical student evaluating a patient with fatigue, fever and joint pain may initially feel anxious, due to the broad range of potential differential diagnoses (stimulus). However, by relying on their training and prior experiences (e.g. moderators) they can adaptively respond—despite their feelings of anxiety (emotional response)—by gathering a history, recommending appropriate tests and consulting with peers and colleagues about the next step to take (behavioural responses) and feeling confident about these next steps in supporting the patient (cognitive response). If this students' uncertainty tolerance was being measured, would they be evaluated as intolerant of uncertainty because of their anxiety or tolerant of uncertainty because of their adaptive behaviours and cognition? This is where the field remains conflicted and where existing scales may be falling short.^7^

*“Learners can feel negatively about an experience of uncertainty but can act adaptively (e.g. with uncertainty tolerance) in response through their cognition and behaviour*.^*6*^ *”*

Most uncertainty tolerance scales were developed and tested using classical test theory (CTT) approaches and prior to the modern multi‐component conceptual model of uncertainty tolerance.^4^ Due to this, existing scales have a tendency towards numerous items focusing on one element of the construct—that of emotional responses and often fail to capture the complex and nuanced nature of the construct.^3^

*“Most uncertainty tolerance scales were developed and tested using classical test theory (CTT) approaches, and prior to the modern multi‐component conceptual model of uncertainty tolerance*.^*4*^ *Due to this, existing scales have a tendency towards numerous items focusing on one element of the construct—that of emotional responses and often fail to capture the complex and nuanced nature of the construct*.^*3*^ *”*

As managing uncertainty becomes increasingly recognised as a health professions' competency,^8^, ^9^, ^10^, ^11^ the desire to measure learners' uncertainty tolerance has equally gained attention—yet multiple studies, including the paper by Wegwarth et al (2025),^1^ call into question the utility of these scales.

## 2 UNCERTAINTY TOLERANCE AND CTT

Psychological and educational measurement instruments are primarily situated within two psychometric worldviews: CTT and latent trait modelling (LTM), such as Item Response Theory (IRT). Both CTT and IRT approaches aim to establish the reliability and validity of scales.^12^ While CTT is the most widely used approach for developing and testing existing uncertainty tolerance scales, it presents significant limitations when applied to assessments of health professions learners' uncertainty tolerance,^13^ and these limitations may contribute to the challenges we have in measuring uncertainty tolerance in this population.

*“While CTT is the most widely used approach for developing and testing existing uncertainty tolerance scales, it presents significant limitations when applied to assessments of health professions learners' uncertainty tolerance,* ^13^ *and these limitations may contribute to the challenges we have in measuring uncertainty tolerance in this population.”*

CTT assumes that an observed score reflects a fixed true score plus random error, with the error considered normally distributed and unrelated to the true score. Under this model, score fluctuations across repeated testing are attributed to external factors—such as test conditions or distractions—rather than actual changes in the trait being measured.^14^ For instance, a student may perform differently on the same test when delivered in a formative assessment context versus under invigilated, high‐stakes conditions. As a result, a scale validated in one context may not maintain its validity in another, requiring revalidation of the same scale in new contexts despite using identical items.

CTT‐based approaches are also sample‐dependent,^15^ meaning reliability and validity estimates are specific to the population in which the scale was developed and may not generalise to other groups. This poses a challenge for applying uncertainty tolerance scales across a diversity of health professions learners. Meta‐analyses of scales developed using CTT methods (e.g. syntheses of Cronbach's alpha) show wide variability in scale performance across populations, necessitating revalidation in each new setting.^2^ Additionally, a scale developed using a CTT approach is not designed to account for context‐driven response differences—such as those between low‐ (e.g. uncertainty with completing a puzzle) and high‐stakes environments (e.g. patient care)—further limiting its ability to provide valid, transferable insights into learners' uncertainty tolerance across contexts.

A further conceptual limitation is the reliance on total scores, which assumes that all items equally reflect the construct, without accounting for item‐level characteristics such as difficulty or discriminatory power—features we consider crucial for measuring the complex construct of uncertainty tolerance. This ‘total score reliance’ limits the sensitivity and precision of existing uncertainty tolerance scales in distinguishing between varying levels of the construct. Uncertainty tolerance often also varies between individuals, even when they encounter the same source of uncertainty. One person may respond confidently to a minor clinical unknown, while another may react with extreme anxiety. This highlights the subjective, dynamic nature of uncertainty tolerance. Scales developed using CTT approaches do not account for differences in test‐takers based on item difficulty or relevance. As a result, instruments developed using CTT approaches are limited in their ability to reflect the nuanced, individualised and varied ways individuals experience and respond to different sources of uncertainty.^15^

## 3 A NEW HORIZON OF UNCERTAINTY TOLERANCE SCALES? THE POTENTIAL ROLE OF LATENT‐TRAIT MODELS

LTMs, including IRT and Rasch models, represent psychometric frameworks that model the relationship between an individual's latent traits and their responses to specific test items. These models estimate the probability of a particular response based on the respondent's underlying trait level and the item characteristics, such as item difficulty and discrimination. Unlike CTT approaches, LTMs offer advantages that make them well‐suited for measuring complex, multidimensional constructs like uncertainty tolerance.

A key advantage of LTMs in measuring constructs like uncertainty tolerance is their emphasis on item‐level analysis rather than reliance on total scores. In the context of uncertainty tolerance, different items may tap into diverse cognitive, emotional or behavioural reactions to uncertainty. LTMs, particularly IRT, allow researchers to evaluate how effectively each item distinguishes between individuals with varying levels of the trait. For example, an item reflecting emotional unease about incomplete information may be highly discriminative among mid‐level uncertainty tolerance respondents but less so at the extremes (those intolerant of uncertainty, for instance). This type of item‐level insight supports more precise scale development opportunities by allowing for retaining items that contribute meaningfully to construct measurement, rather than assuming all items are equally informative.

Another strength of LTMs is their ability to produce sample‐independent, context‐transferable measures. Once items are calibrated using IRT or Rasch analysis with good model fit, their psychometric properties remain stable across populations and settings. This is valuable for developing uncertainty tolerance assessments applicable across diverse health professions learners. For instance, a scale developed with Australian medical students may be extended to physiotherapy or nursing students in other countries. However, cross‐group validation—including tests for measurement invariance and differential item functioning—remains essential to ensure fair and accurate interpretation.^16^

LTMs allow for nuanced insights into individual responses to different sources of, for instance, uncertainty by estimating each respondent's position on the latent trait continuum in a manner that is independent of the test form. For instance, two learners may give the same response to an item on diagnostic ambiguity, but IRT can reveal meaningful differences in overall tolerance based on scale response patterns. This precision supports identifying subgroups and tailoring feedback or interventions. Given the nuanced nature of uncertainty tolerance responses—when emotional, cognitive and behavioural aspects of uncertainty tolerance vary across individuals—this could prove valuable in the field.

We encourage uncertainty tolerance researchers to consider LTM holistically from conception and design of the uncertainty tolerance scale through to evaluation. In this phase of transition, however, we suggest that researchers explore existing uncertainty tolerance scales, often developed through CTT approaches, with LTM evaluation strategies.

*“We encourage uncertainty tolerance researchers to consider LTM holistically from conception and design of the uncertainty tolerance scale through to evaluation.”*

## 4 THE FUTURE OF UNCERTAINTY TOLERANCE SCALES

“You cannot swim for new horizons until you have courage to lose sight of the shore”—William Faulkner.

As William Faulkner suggests, the recent paper by Wegworth and colleagues^1^ can serve as a ‘north star’ that inspires the field to lose sight of the shore (e.g. uncertainty tolerance scales developed and evaluated using CTT approaches) and move in the direction of a new horizon (e.g. development and evaluation of these scales using LTM approaches). When we take a closer look at the historical uncertainty tolerance scale “shore”, we see some significant challenges. These include a primary focus on measuring one feature of the construct (i.e. emotional responses), as well as the inability to tease out potential moderating factors (e.g. related to individual items) influencing an individual's perception and responses to uncertainty.^17^, ^18^, ^19^

*‘“You cannot swim for new horizons until you have courage to lose sight of the shore”—William Faulkner. As William Faulkner suggests, the recent paper by Wegworth and colleagues* ^*1*^ *can serve as a ‘north star’ that inspires the field to lose sight of the shore (e.g. uncertainty tolerance scales developed and evaluated using CTT approaches) and move in the direction of a new horizon (e.g. development and evaluation of UT scales using LTM approaches).’*

Given advances in measurement theory applicable to health professions education, there is an opportunity now to adjust our course towards a new horizon of LTM. LTM approaches could consider known relevant features of uncertainty tolerance including the emotional, cognitive and behavioural elements as well as the moderating factors which may be influencing an individual learners' response to uncertainty—and even consider how different sources of uncertainty may play a role in these responses. In this way, LTM approaches offer a way to advance the measurement of this complex construct.

Given the widespread inclusion of uncertainty tolerance as a health profession graduate attribute and the desire to measure uncertainty tolerance across health professions, there is a clear imperative to embrace more nuanced psychometric approaches. The assumptions of LTM demonstrate congruence with the modern conceptual uncertainty tolerance modelling and could allow for scale development and measurement which are theoretically grounded, empirically robust and responsive to the realities of modern health professions education. The field is ready to lose sight of the shore and swim for new horizons.

## AUTHOR CONTRIBUTIONS

**Michelle D. Lazarus:** Conceptualisation; writing—original draft; writing—review and editing. **Melanie Farlie:** Conceptualisation; writing—original draft; writing—review and editing. **Md Nazmul Karim:** Conceptualisation; writing—original draft; writing—review and editing.
